# Gambling and its association with psychological distress among different population groups: a cross-sectional study in Great Britain, 2022

**DOI:** 10.1186/s12889-026-27630-8

**Published:** 2026-05-06

**Authors:** Sarah E. Jackson, Jamie Brown, Melissa Oldham, Lion Shahab, Loren Kock

**Affiliations:** 1 Department of Behavioural Science and Health, University College London, 1-19 Torrington Place, London, WC1E 7HB UK; 2 SPECTRUM Consortium, Edinburgh, UK

**Keywords:** Gambling, Psychological distress, Mental health

## Abstract

**Objectives:**

To estimate associations between past-year gambling and risk of harm from gambling with psychological distress, and explore whether these associations differ across population subgroups.

**Methods:**

Data were collected from 1,987 adults (≥ 18y) in Great Britain in October 2022 through a nationally-representative household survey. Participants reported past-year gambling activity. Those who had gambled completed the Problem Gambling Severity Index; scores ≥ 1 indicated the person was at risk of harm from gambling. Past-30-day psychological distress was assessed with the K6 and categorised as low/no (scores < 5) vs. moderate/severe distress (≥ 5). Covariates included sociodemographics, mental health history, smoking status, and alcohol consumption.

**Results:**

Past-year gambling was not associated with increased odds of experiencing psychological distress relative to not gambling (29.7% vs. 32.6%; OR = 0.87 [0.70–1.09]; OR_adj_=0.81 [0.62–1.06]), and there was no clear evidence of moderation by age, gender, social grade, children in the household, mental health history, smoking, or alcohol consumption. Among past-year gamblers, those at risk of harm from gambling were more likely to report distress than those not at risk (46.9% vs. 28.2%; OR = 2.25 [1.23–4.10]). This association was attenuated after adjustment for sociodemographics (OR_adj_=1.67 [0.84–3.35]) and additionally for mental health history, smoking status, and alcohol consumption (OR_adj_=1.16 [0.57–2.35]).

**Conclusions:**

Psychological distress appears similar between those who had and had not gambled in the past year in Great Britain in 2022. Those at risk of harm from gambling were more likely to experience psychological distress but it does not appear independent of their sociodemographic characteristics, mental health history, and smoking and alcohol consumption. These findings underscore the need for integrated policies and intervention strategies that address not only gambling behaviour but also consider associated sociodemographic and health-related factors to effectively mitigate distress among individuals at risk.

**Supplementary Information:**

The online version contains supplementary material available at 10.1186/s12889-026-27630-8.

## Introduction

Gambling is a prevalent activity across many societies, with implications for public health and wellbeing [1]. In Great Britain, gambling is legal for those aged ≥ 18 and approximately half of all adults gamble each year [2, 3]. While many people engage in gambling as a recreational activity without adverse consequences, for some it can lead to serious problems including financial hardship, relationship issues, and poor mental health [1, 4]. The 2023 Gambling Survey for Great Britain found that 23.9% of adults in Great Britain who gambled in the past year were at risk of experiencing harm as a result of their gambling, according to the Problem Gambling Severity Index (PGSI score ≥ 1) [5]. While most of those with a PGSI score ≥ 1 scored at the lower end of the spectrum (between 1 and 3; 13.7% of past-year gamblers), some had very high scores, with 0.4% of all past-year gamblers scoring 20 or above [5]. These issues have prompted considerable debate and the need for research to better understand the scope of gambling-related harm [6].

One important aspect of gambling-related harm is its relationship with mental health. This includes long-term impacts such as persistent mental health conditions as well as more acute impacts such as the experience of psychological distress (which may occur outside of a diagnosable mental health condition). Previous studies have shown that people who engage in gambling are at increased risk of psychological distress [7–9]. In particular, those who gamble more frequently or whose gambling leads to adverse consequences (e.g., financial or relationship problems) tend to be more susceptible to experiencing mental health concerns [10–13], often as a result of debt or financial problems [14]. This can lead to a vicious cycle of gambling and poor mental health, as gamblers who experience distress are more likely to exhibit behaviours that further exacerbate their mental health problems, such as further gambling or substance misuse [8, 15].

While studies consistently find gambling to be associated with psychological distress [7–9], the evidence base is limited by a lack of representative population-based studies. In addition, the extent to which associations between gambling and psychological distress vary across population subgroups is unclear. Certain groups may be more vulnerable to the risks of gambling and its associated mental health effects, due to factors such as age, gender, socioeconomic position, family responsibilities, pre-existing mental health conditions, and other addictive behaviours (e.g., tobacco and alcohol use). Understanding the prevalence of gambling and its potential impact on psychological distress among different population groups can inform public health strategies, regulatory frameworks, and support services aimed at reducing gambling-related harm.

In Great Britain, the need for comprehensive, recent information on gambling and its relationship with psychological distress within different population groups is particularly pressing. The country has been experiencing a ‘cost-of-living crisis’ since late 2021, with high rates of inflation causing the cost of everyday essentials like food and utility bills to increase more quickly than the average household income, putting pressure on household budgets – particularly among less socioeconomically advantaged groups [16]. Gambling retailers tend to be concentrated in deprived areas, so may disproportionately affect less advantaged groups [17, 18]. In addition, there has been a considerable rise in the proportion of adults experiencing psychological distress over this period – especially among younger adults [19]. The impacts of the cost-of-living and mental health crises have not been experienced equally across the population: for example, people from less socioeconomically advantaged groups have been less able to cope financially in the face of high rates of inflation [20, 21], while younger age groups have seen particularly large increases in the prevalence of psychological distress [19]. It is therefore particularly important to examine these relationships in the current context, where financial pressures and psychological distress are heightened across the population.

Using data from a nationally representative survey of adults in Great Britain in 2022, this study aimed to provide descriptive data on the prevalence of gambling, estimate associations between gambling and psychological distress, and explore differences across population subgroups. Specifically, we aimed to:

1. Estimate the extent to which (i) past-year gambling and (ii) being at any risk of harm from gambling are associated with experiencing moderate or severe past-30-day psychological distress.
2. Explore whether the association between past-year gambling and psychological distress differs by age, gender, occupational social grade, children in the household, history of mental health conditions, smoking status, and level of alcohol consumption.

Additionally, we extrapolated prevalence estimates to the adult population in Great Britain to provide approximate numbers of adults engaging in gambling and those at risk of gambling-related harm, offering context for the potential public health impact.

## Methods

### Pre-registration

The study protocol and analysis plan were pre-registered on Open Science Framework (https://osf.io/yqrj4/).

### Design

Data were collected via the Smoking and Alcohol Toolkit Study, a monthly cross-sectional survey of a representative sample in Great Britain [22, 23]. The study uses a hybrid sampling method that combines random probability with simple quota sampling to recruit a new sample of around 2,450 people aged ≥ 16 years each month. Data are gathered through computer-assisted telephone interviews. Comparisons with other national surveys and sales data show that core variables, including sociodemographic characteristics, are representative of the national population [22]. The core interview is available on www.smokinginengland.info. In October 2022, questions on gambling were added to the survey for a single wave. These questions were asked to all participants aged ≥ 18y in England and ~ 50% of participants in Wales and Scotland (due to limited research funding). The present study analysed data from participants surveyed in this wave who provided data on gambling.

### Measures

#### Past-year gambling

Past-year gambling was defined as reporting having engaged in any of the following gambling types in the past 12 months: national lottery, other lotteries, or scratch cards; football pools; bingo (not online); slot machines; machines in a bookmakers; casino table games (not online); online gambling in slots, casino, or bingo; online betting with a bookmaker; betting exchange; horse races (not online); dog races (not online); sports events (not online); private betting; loot boxes or skins gambling within online/video games; crypto casinos; any other gambling event or activity. As a sensitivity analysis, we excluded exclusive lottery gambling (i.e., reporting only engaging in ‘national lottery, other lotteries, or scratch cards’) from the definition of past-year gambling, given this has previously been shown to be the most prevalent and least harmful type of gambling [24].

#### Risk of harm from gambling

Among those who reported having gambled in the past year (including exclusive lottery gambling), problem gambling was assessed with the Problem Gambling Severity Index (PGSI). This is a nine-item questionnaire, derived from the Canadian Problem Gambling Index [25], which asks: ‘Thinking about the last 12 months…’.

1. Have you bet more than you could really afford to lose?
2. Have you needed to gamble with larger amounts of money to get the same feeling of excitement?
3. When you gambled, did you go back another day to try to win back the money you lost?
4. Have you borrowed money or sold anything to get money to gamble?
5. Have you felt that you might have a problem with gambling?
6. Has gambling caused you any health problems, including stress or anxiety?
7. Have people criticized your betting or told you that you had a gambling problem, regardless of whether or not you thought it was true?
8. Has your gambling caused any financial problems for you or your household?
9. Have you felt guilty about the way you gamble or what happens when you gamble?

For each item the response options are never/none of the time (scored 0), sometimes (1), most of the time (2), and almost always (3). Scores for each item are summed to create a total score of 0–27. The scale had good internal consistency (Cronbach’s α = 0.89). Scores can be classified as no risk (0), low-risk (1–4), moderate risk (5–7), or problem gambling (≥ 8). For our analyses, we distinguished between those scoring ≥ 1 (any risk) vs. 0 (no risk). Values were imputed as 0 for those who did not report past-year gambling.

#### Psychological distress

Psychological distress was measured using the Kessler Psychological Distress Scale (K6), which assesses general psychological distress experienced over the past 30 days [26, 27]. The scale includes six items asking how often respondents felt: (a) nervous; (b) hopeless; (c) restless or fidgety; (d) so depressed that nothing could cheer you up; (e) that everything was an effort; (f) worthless?’ Each item is rated on a 5-point scale ranging from 0 (none of the time) to 4 (all of the time), with total scores ranging from 0 to 24. The scale had good internal consistency (Cronbach’s α = 0.87). A score of 0 indicates no distress, 1–4 indicates low distress, 5–12 moderate distress, and 13–24 severe distress [26, 28]. For analysis, scores were dichotomised into no/low distress (0–4) and moderate/severe distress (≥ 5).

#### Participant characteristics

Age was analysed as a continuous variable. Gender was categorised as identifying as a man, woman, or in another way. Those who identified in another way were excluded from analyses by gender due to low numbers. Occupational social grade was categorised according to the National Readership Survey classification [29] as AB (higher and intermediate managerial, administrative, and professional); C1 (supervisory, clerical and junior managerial, administrative, and professional); C2 (skilled manual workers); D (semi-skilled and unskilled manual workers); or E (state pensioners, casual and lowest grade workers, unemployed with state benefits only). Children in the household was categorised as yes or no.

History of mental health conditions was based on self-reported diagnosis of at least one mental or behavioural disorder (e.g., depression, anxiety, post-traumatic stress disorder) since the age of 16 (yes/no). Smoking status was categorised as current, former, or never smoker. Level of alcohol consumption was assessed with the Alcohol Use Disorders Identification Test—consumption (AUDIT-C), with possible scores ranging from 0 to 12. As a general guide, scores ≥ 5 indicate drinking at increasing or higher-risk levels (i.e., levels that increase someone’s risk of harm) [30].

### Statistical analysis

Data were analysed in R v 4.2.2. We excluded participants with missing data on past-year gambling; missing cases on other variables were excluded on a per-analysis basis. The Smoking and Alcohol Toolkit Study uses raking to weight the sample to match the population of Great Britain in terms of key demographics [22]. Weights take into account gambling variables not being assessed among all participants in Wales and Scotland.

We reported the proportions (with 95% confidence interval [CI]) reporting past-year gambling and, among those who had gambled in the past year, risk of harm from gambling, among all adults and by each participant characteristic. We used mid-year population estimates for Great Britain [31] to extrapolate these proportions to estimate the corresponding number of adults affected at the population level. Age and AUDIT-C score were modelled non-linearly using restricted cubic splines (with three knots placed at the 5, 50, and 95% quantiles for age and at scores of 0, 6, and 12 for AUDIT-C) to allow for flexible associations without arbitrary categorisation. Estimates were predicted from unadjusted logistic regression models that tested associations of age and AUDIT-C score with the gambling outcomes. To aid interpretation, we report estimates for selected ages (18, 25, 35, 45, 55, and 65) and AUDIT-C scores (0, 3, 6, 9, and 12), which were chosen to illustrate differences across the spectrum of ages and alcohol consumption levels rather than to imply fixed categories. This approach avoids loss of information that can occur when continuous variables are grouped, while still providing interpretable estimates for the reader. To improve transparency, we also provide supplementary plots of the full predicted relationships between age and AUDIT-C score with gambling outcomes so that readers can see how results vary across all values.

We used logistic regression to test associations of (i) past-year gambling (including and excluding exclusive lottery gambling) and (ii) being at risk of any harm from gambling with psychological distress (no/low distress [ref] vs. moderate/severe distress), with and without adjustment for age, gender, occupational social grade, children in the household, history of mental health conditions, smoking status, and level of alcohol consumption. As a sensitivity analysis, we repeated these models without adjustment for history of mental health conditions, smoking status, and level of alcohol consumption as these could potentially be, at different points in time, a cause or a consequence of psychological distress related to gambling [32, 33]. We then repeated the fully adjusted models removing each of these variables in turn.

We then repeated the adjusted models testing interactions between past-year gambling (including and excluding exclusive lottery gambling) and each participant characteristic on distress, to explore whether associations between gambling and distress differed across subgroups. Each interaction was tested in a separate model.

## Results

A total of 2,398 people aged ≥ 16 years in Great Britain responded to the survey in October 2022. We excluded 25 participants aged 16–17 years and a further 364 participants in Wales and Scotland who were not asked the questions on gambling. We also excluded 22 participants who either did not respond to the question assessing past-year gambling or who responded that they did not know. This left an analytic sample of 1,987 participants. The sample had a weighted mean age of 48.3 years and 51.2% were women. Table S1 provides a summary of the sample characteristics and missing data on each variable.

### Prevalence of past-year gambling

Table 1 shows the prevalence of past-year gambling, overall and within subgroups. Predicted estimates for each level of age and AUDIT-C are shown in Figure S1. Overall, 48.2% of participants reported past-year gambling. The majority (58.4% [54.9–61.8%]) of this group only engaged in lottery gambling; prevalence of past-year gambling was 20.0% excluding those who reported exclusive lottery gambling. This equates to approximately 25.4 million past-year gamblers (52.7 million adults ≥ 18y * 48.2%); 10.5 million excluding exclusive lottery gamblers (52.7 million * 20.0%).

**Table 1:** Prevalence of past-year gambling and risk of harm from gambling among adults in Great Britain

	Prevalence, % [95%CI]				
	Past-year gambling		Risk of harm from gambling		
	Including ELG	Excluding ELG	Among adults	Among past-year gamblers (including ELG)	Among past-year gamblers (excluding ELG)
All adults	48.2 [45.7–50.6]	20.0 [18.1–22.0]	3.3 [2.4–4.2]	6.9 [5.0–8.8]	13.3 [9.4–17.2]
Age (years)^1^					
18	32.3 [26.0–39.4]	27.9 [21.5–35.3]	6.0 [3.1–11.3]	17.7 [9.4–30.9]	21.1 [10.3–38.2]
25	41.3 [36.7–46.1]	26.3 [22.3–30.7]	5.1 [3.4–7.7]	12.6 [8.4–18.5]	17.2 [11.1–25.7]
35	50.9 [47.6–54.1]	24.3 [21.6–27.3]	4.3 [3.0–6.0]	8.6 [6.1–11.9]	13.8 [9.5–19.6]
45	56.7 [53.2–60.2]	21.9 [19.0–25.0]	3.5 [2.3–5.3]	6.1 [3.9–9.3]	11.4 [6.9–18.3]
55	56.3 [52.8–59.8]	18.6 [16.1–21.4]	2.7 [1.7–4.0]	4.7 [3.0–7.1]	10.3 [6.3–16.5]
65	49.6 [46.4–52.9]	14.9 [12.7–17.4]	1.9 [1.2–3.1]	4.0 [2.4–6.4]	10.2 [6.1–16.6]
Gender					
Men	51.3 [47.9–54.8]	25.4 [22.3–28.4]	4.9 [3.3–6.5]	9.6 [6.5–12.6]	17.3 [11.6–22.9]
Women	45.3 [41.8–48.8]	15.3 [12.8–17.9]	1.6 [0.7–2.6]	3.7 [1.6–5.7]	6.5 [2.0–11.1]
Occupational social grade					
AB (most advantaged)	46.4 [42.0–50.8]	17.9 [14.5–21.3]	2.1 [0.9–3.4]	4.6 [1.9–7.4]	9.9 [3.6–16.3]
C1	49.1 [45.6–52.6]	19.4 [16.6–22.1]	2.3 [1.2–3.4]	4.7 [2.6–6.9]	9.2 [4.4–14.0]
C2	49.6 [43.8–55.5]	22.2 [17.3–27.0]	2.9 [1.1–4.8]	5.9 [2.2–9.6]	8.8 [2.2–15.4]
D	49.4 [40.8–58.1]	24.1 [16.6–31.5]	7.9 [3.4–12.3]	15.9 [7.2–24.5]	27.3 [11.4–43.2]
E (least advantaged)	45.1 [36.3–53.8]	17.6 [10.8–24.4]	3.5 [0.2–6.8]	8.0 [0.5–15.4]	20.2 [1.7–38.7]
Children in the household					
No	47.1 [44.3–50.0]	18.9 [16.6–21.2]	3.1 [2.0–4.1]	6.5 [4.4–8.7]	13.0 [8.3–17.7]
Yes	50.8 [46.2–55.4]	22.9 [19.0–26.9]	3.9 [2.0–5.8]	7.7 [4.0–11.4]	13.9 [6.7–21.1]
History of ≥ 1 mental health conditions					
No	46.7 [43.8–49.5]	19.1 [16.8–21.4]	2.5 [1.5–3.4]	5.3 [3.3–7.3]	10.4 [6.1–14.7]
Yes	51.5 [46.9–56.1]	22.1 [18.3–26.0]	5.2 [3.1–7.3]	10.1 [6.2–14.1]	18.9 [10.9–26.9]
Smoking status					
Never	43.4 [40.3–46.5]	17.0 [14.6–19.4]	2.3 [1.3–3.3]	5.4 [3.1–7.7]	11.1 [6.0–16.2]
Former	54.5 [49.6–59.4]	21.0 [16.9–25.1]	3.9 [1.9–5.9]	7.2 [3.6–10.9]	13.6 [5.6–21.6]
Current	54.2 [47.7–60.7]	28.2 [22.4–34.1]	5.3 [2.4–8.1]	9.8 [4.5–15.0]	16.1 [7.0–25.2]
Level of alcohol consumption (AUDIT-C)^2^					
0 (lowest)	37.4 [33.2–41.8]	11.8 [9.1–15.1]	2.0 [1.1–3.7]	5.5 [3.0–9.9]	9.5 [4.6–18.8]
3	49.7 [46.7–52.7]	19.2 [17.0–21.6]	3.7 [2.7–5.2]	7.6 [5.4–10.6]	15.3 [10.5–21.9]
6	57.4 [53.7–61.1]	27.2 [23.6–31.0]	4.7 [3.2–7.0]	8.2 [5.5–12.0]	16.6 [11.1–24.1]
9	57.4 [51.2–63.4]	32.3 [26.8–38.3]	3.3 [1.6–6.6]	5.7 [2.7–11.4]	9.4 [4.1–20.1]
12 (highest)	52.7 [38.3–66.6]	35.0 [22.4–50.0]	1.6 [0.3–9.0]	3.0 [0.5–16.9]	3.4 [0.4–24.8]

*CI* Confidence interval, *ELG* Exclusive lottery gambling

^1^ Predicted estimates from a logistic regression model with age modelled using restricted cubic splines. Note that the model used to derive these estimates included data from participants of all ages, not only those who were aged exactly 16, 25, 35, 45, 55, or 65 years. Predicted estimates for each level of age are shown in Figure S1

^2^ Predicted estimates from a logistic regression model with AUDIT-C score modelled using restricted cubic splines. Note that the model used to derive these estimates included data from all participants who provided data on AUDIT-C, not only those who scored exactly 0, 3, 6, 9, or 12. Predicted estimates for each level of AUDIT-C are shown in Figure S1

Groups more likely to report past-year gambling, especially excluding exclusive lottery gambling, included men, people who currently smoked (vs. never smoked), and people with higher (vs. lower) levels of alcohol consumption. Prevalence of any past-year gambling was higher among middle-aged and older adults (≥ 35 years) compared with younger adults (< 35 years), but prevalence of non-lottery gambling was higher at younger ages. There were no notable differences by occupational social grade, presence of children in the household, or history of mental health conditions (as indicated by overlapping 95% CIs).

### Prevalence of risk of harm from gambling

Table 1 shows the prevalence of risk of harm from gambling, overall and within subgroups. Overall, 3.3% of participants were at risk of harm from gambling; approximately 1.7 million adults (52.7 million * 3.3%). This included 6.9% of past-year gamblers and 13.3% of non-lottery gamblers.

Among adults overall and among past-year gamblers (including and excluding exclusive lottery gamblers), groups more likely to be at risk of harm from gambling included younger adults, men, people from less advantaged social grades, and people who smoked. The proportions at risk of harm from gambling were also approximately twice as high among those with vs. without a history of mental health conditions, although there was some overlap in the 95% CIs around these estimates. Differences by level of alcohol consumption did not follow a clear pattern, but prevalence appeared slightly higher among those who drank at low to moderate levels (AUDIT-C: 3–6). There were no notable differences according to the presence of children in the household.

### Associations of gambling with psychological distress

**Fig. 1:**
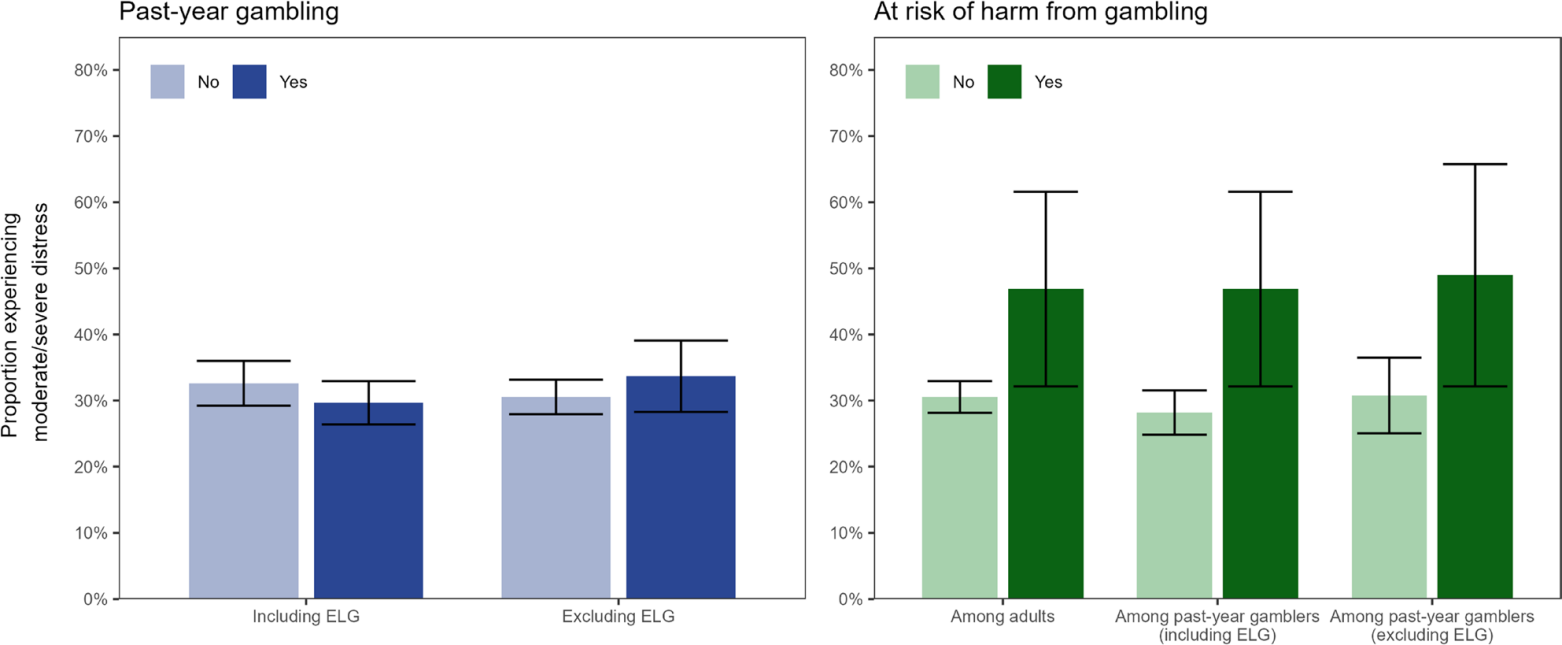
Prevalence of psychological distress in relation to past-year gambling and risk of harm from gambling. ELG, exclusive lottery gambling. Bars show the weighted proportions of adults in Great Britain reporting no/low distress and moderate/severe distress. Error bars represent 95% confidence intervals

Overall, 31.2% [28.8–33.5%] of participants reported experiencing moderate/severe psychological distress. There was no notable difference in psychological distress between those who had and had not gambled in the past year (Fig. 1; Table 2). This pattern held when we restricted the definition of past-year gambling to exclude exclusive lottery gambling. There was no clear evidence that the association between past-year gambling (including or excluding exclusive lottery gambling) and psychological distress was moderated by age, gender, occupational social grade, children in the household, history of mental health conditions, smoking status, or level of alcohol consumption (Table S2).

**Table 2:** Associations of gambling with past-month psychological distress

	Moderate/severe distress			
	% [95% CI]^1^	OR [95%CI]^2^	OR_adj_ [95%CI]^3^	Sensitivity OR_adj_ [95%CI]^4^
Past-year gambling (including ELG)				
No	32.6 [29.2–36.0]	-	-	-
Yes	29.7 [26.4–32.9]	0.87 [0.70–1.09]	0.81 [0.62–1.06]	0.92 [0.72–1.17]
Past-year gambling (excluding ELG)				
No	30.5 [27.9–33.2]	-	-	-
Yes	33.7 [28.3–39.1]	1.15 [0.88–1.51]	0.87 [0.63–1.21]	0.92 [0.68–1.24]
Risk of harm from gambling (among adults)				
No	30.5 [28.1–32.9]	-	-	-
Yes	46.9 [32.2–61.6]	2.01 [1.11–3.61]	0.97 [0.48–1.95]	1.43 [0.73–2.81]
Risk of harm from gambling (among past-year gamblers, including ELG)				
No	28.2 [24.8–31.5]	-	-	-
Yes	46.9 [32.1–61.6]	2.25 [1.23–4.10]	1.16 [0.57–2.35]	1.67 [0.84–3.35]
Risk of harm from gambling (among past-year gamblers, excluding ELG)				
No	30.8 [25.0–36.5]	-	-	-
Yes	49.0 [32.2–65.8]	2.16 [1.07–4.38]	1.25 [0.50–3.10]	2.23 [1.01–4.92]

*CI* Confidence interval, *ELG* Exclusive lottery gambling, *OR*_adj_ Adjusted odds ratio

^1^ Unadjusted prevalence of moderate/severe distress

^2^ Estimates from unadjusted logistic regression

^3^ Estimates from logistic regression adjusted for age, gender, occupational social grade, children in the household, history of ≥ 1 mental health conditions, smoking status, and level of alcohol consumption

^4^ Estimates from logistic regression adjusted for age, gender, occupational social grade, and children in the household

Those who were at risk of harm from gambling were more likely to report psychological distress than those who were not (Fig. 1). In unadjusted models, the odds of psychological distress were around twice as high for those at risk of harm from gambling, compared with adults and past-year gamblers who were not at risk of harm from gambling (Table 2). These differences were attenuated after adjusting for sociodemographic characteristics, history of mental health conditions, smoking status, and level of alcohol consumption (Table 2). Sensitivity analyses that only adjusted for sociodemographic characteristics showed more pronounced differences compared with the fully adjusted model, suggesting a considerable part of the association between risk of harm from gambling and psychological distress may have been explained by history of mental health conditions, smoking status, and/or level of alcohol consumption. Removing these factors from the fully adjusted model separately in turn suggested history of mental health conditions was most influential, while smoking and alcohol consumption had smaller effects (Table S3), indicating that overlapping vulnerabilities, particularly pre-existing mental health conditions, substantially contribute to the observed association.

## Discussion

In October 2022, around one in two adults in Great Britain reported having gambled in the past year and one in five had engaged in non-lottery gambling. One in 30 adults – around one in seven past-year gamblers – was classified at risk of harm from their gambling according to the PGSI which covers a range of potential harms including financial problems, relationship damage and mental health. Past-year gambling was not associated with increased odds of experiencing psychological distress relative to not gambling. However, among past-year gamblers, those at risk of harm from gambling were more likely to report distress than those not at risk. This association was attenuated after adjustment for sociodemographic characteristics, history of mental health conditions, smoking, and alcohol consumption, but persisted after adjustment for sociodemographic characteristics alone when the sample was restricted to past-year gamblers who engaged in non-lottery gambling.

It is important to note that the PGSI was originally developed to assess problem gambling rather than the broader concept of gambling-related harm [25]. While the PGSI captures a range of individual-level harms associated with gambling behaviour, such as financial problems, emotional distress, and loss of control, it does not fully assess wider consequences of gambling, including impacts on family members, friends, or the community. Consequently, our estimates of harm do not take into account the broader societal and relational impacts of gambling and are therefore likely to underestimate the extent of the problem. Future research could complement the PGSI with additional indicators or measures that capture this wider spectrum of gambling-related harm, to provide a more comprehensive picture of its public health implications.

Consistent with previous studies [34–36], we observed variation across subgroups. Younger adults, men, people who smoked, and people with higher levels of alcohol consumption were more likely to report non-lottery gambling. In addition, younger adults, men, people from less advantaged social grades, people with a history of mental health conditions, and people who smoked were more likely to be at risk of harm from gambling. These subgroup differences may be the result of various behavioural, social, and psychological factors. Younger adults are more prone to impulsive decisions than those who are older [37] and have greater exposure to online gambling platforms, which are highly accessible and addictive [38]. Men tend to engage in more risk-taking behaviour than women [39] and are more likely to gamble for excitement and thrill seeking, often wagering larger amounts [40]. People from less advantaged social grades may gamble in an attempt to improve their financial situation [41], a behaviour that may be exacerbated by targeted marketing from gambling companies [17, 18]. For people with a history of mental health conditions, gambling may serve as a maladaptive coping mechanism [42] and can exacerbate existing emotional distress [8, 15]. People who smoke appear to have higher susceptibility to other addictive behaviours [35, 43] and may have higher exposure to environments that promote gambling (e.g., casinos or bars) [35], reinforcing risky behaviour. Both mental health challenges and smoking influence reward systems in the brain, making it harder to break cycles of harmful gambling [44].

Our findings are also consistent with previous research linking gambling-related harm to poorer mental health outcomes. Studies have found that frequent gambling and gambling-induced financial or relational problems are linked to psychological distress, often creating a cycle of harm that exacerbates both gambling behaviours and mental health outcomes [8, 10–13, 15]. Our data show people at risk of harm from gambling – particularly those who engaged in non-lottery gambling – were more likely to report psychological distress than those not at risk. The relationship attenuated after adjustment for sociodemographic characteristics, mental health history, smoking, and alcohol consumption, suggesting that the observed association may be partly explained by these overlapping vulnerabilities. The absence of an association between past-year gambling and distress in this study may reflect the inclusion of less harmful forms of gambling, such as lottery gambling, which accounts for a substantial portion of gambling behaviour but is less strongly associated with harm [24]. This distinction underscores the importance of disaggregating gambling types in research on its impacts.

We had expected that any association between gambling and psychological distress might be more pronounced among groups experiencing greater disadvantage (e.g., people from less advantaged social grades) or those more susceptible to distress (e.g., people with a history of mental health conditions). However, our results showed no clear evidence that the (null) association between past-year gambling and distress differed across population subgroups. We did not explore moderation of the association between risk of harm from gambling and distress because small sample sizes meant we would likely have been underpowered to detect differences. It is therefore possible that risk of harm from gambling may be more strongly associated with psychological distress among certain subgroups, which requires further exploration.

Our findings have implications for public health. The numbers at risk of harm reinforces the need for a population-level approach combined with targeted policy to groups identified as more vulnerable, such as younger adults, men, and people from less advantaged occupational social grades. While the 2023 gambling White Paper *High Stakes: gambling reform for the digital age* proposes a number of regulatory changes including financial risk checks, maximum bets, and advertising restrictions [45], researchers have argued that these measures do not go far enough [46, 47]. The attenuation of associations between gambling harm and psychological distress after adjusting for smoking, alcohol consumption, and history of mental health conditions suggests that interventions addressing these overlapping factors may have synergistic benefits. This approach may be especially important in the current context of economic strain, which may be exacerbating financial, mental health, and substance use problems [48–50]. In light of this, there is a need to consider more integrated policies and intervention strategies that not only reduce harmful gambling behaviours but also tackle the wider social and health determinants – such as economic disadvantage [51], co-occurring substance use [52], and pre-existing mental health conditions [53] – that can heighten vulnerability and perpetuate cycles of distress. Such approaches, spanning prevention, early intervention, and treatment, may offer greater effectiveness than gambling-focused policies alone.

Key strengths of this study were the nationally representative sample and detailed measures of gambling behaviour, psychological distress, and relevant sociodemographic and behavioural factors. There were also limitations. The reliance on self-reported measures may introduce bias, particularly in the context of sensitive issues such as gambling and mental health. The cross-sectional design meant that we could not determine the direction of causality between risk of harm from gambling and psychological distress (i.e., whether risky gambling contributes to distress or distress makes people more likely to engage in risky gambling). However, we note the measure assessed experiences of distress over the past 30 days, which may not capture underlying long-term distress and thus may be confounded by acute factors not considered here (e.g., recent stressful life events). The small sample sizes for certain subgroups limited statistical power, so there may be differences that we were unable to detect. In addition, there may also be intersectional subgroups among whom the proportion at risk of harm from gambling is particularly high (e.g., young men from less advantaged occupational social grades); this would be interesting to explore with a larger sample. Finally, while the survey was conducted during a period of heightened financial pressure due to the cost-of-living crisis, the extent to which these broader economic conditions influenced gambling behaviours and mental health outcomes remains an area for further research.

In conclusion, in 2022, around half adults in Great Britain had gambled in the past year and one in 30 were at risk of harm from gambling. Those at risk of harm from gambling were more likely to experience psychological distress. This association appeared to be largely explained by sociodemographic characteristics, history of mental health conditions, smoking, and alcohol consumption.

## Supplementary Information

Supplementary Material 1

## Data Availability

Data used in these analyses are available on Open Science Framework ( [https://osf.io/yqrj4/](https:/osf.io/yqrj4) ), with age provided in bands to preserve anonymity.

## Abbreviations

PGSI: Problem Gambling Severity Index
