# Supplementary material for: Gambling and its association with psychological distress among different population groups: a cross-sectional study in Great Britain, 2022
Source: BMC Public Health. 2026 May 6;26:1959. doi: 10.1186/s12889-026-27630-8 (PMC13312683; doi:10.1186/s12889-026-27630-8)
Supplement: Supplementary file 1 — Supplementary Material 1 [file 12889_2026_27630_MOESM1_ESM.pdf]

Table S1. Weighted sample characteristics

	**Unweighted *n***	**Weighted %[1]**
**All adults**	1987	100
Age, mean (SD)	1987	48.3 (18.4)
Missing	0	-
Gender		
Men	968	48.4%
Women	994	51.2%
Non-binary	8	0.4%
Missing	17	-
Occupational social grade		
AB (most advantaged)	535	26.3%
C1	860	29.8%
C2	310	20.4%
D	145	14.3%
E (least advantaged)	137	9.3%
Missing	0	-
Children in the household		
No	1432	71.5%
Yes	555	28.5%
Missing	0	-
History of ≥1 mental health conditions		
No	1414	69.1%
Yes	573	30.9%
Missing	0	-
Smoking status		
Never smoker	1182	58.8
Former smoker	504	25.0
Current smoker	283	16.2
Missing	18	-
Level of alcohol consumption, mean (SD)		
AUDIT-C score	1929	3.46 (2.98)
Missing	58	-
Psychological distress		
No/low	1395	68.8%
Moderate	419	22.9%
Severe	127	8.3%
Missing	46	-
Past-year gambling	1987	48.2%
Missing	0	-
Risk of harm from gambling	1975	3.3%
Missing	12	-

[1] Data are shown as weighted percentages, unless otherwise specified. Sample sizes (including numbers of missing cases) are unweighted.

Table S2. Moderation of associations of past-year gambling with psychological distress by participant characteristics

	Including ELG		Excluding ELG	
	**F**	***p***	**F**	***p***
Age	0.10	0.902	0.51	0.597
Gender	0.10	0.752	3.20	0.074
Occupational social grade	1.77	0.133	0.21	0.933
Children in the household	0.10	0.747	<0.01	0.987
History of ≥1 mental health conditions	<0.01	0.997	0.99	0.319
Smoking status	1.16	0.313	0.23	0.798
Level of alcohol consumption	2.32	0.099	1.30	0.272

ELG, exclusive lottery gambling.
Wald test for the interaction between each characteristic and past-year gambling in models testing associations with psychological distress (moderate/severe vs. no/low distress), adjusted for age, gender, occupational social grade, children in the household, history of ≥1 mental health conditions, smoking status, and level of alcohol consumption.

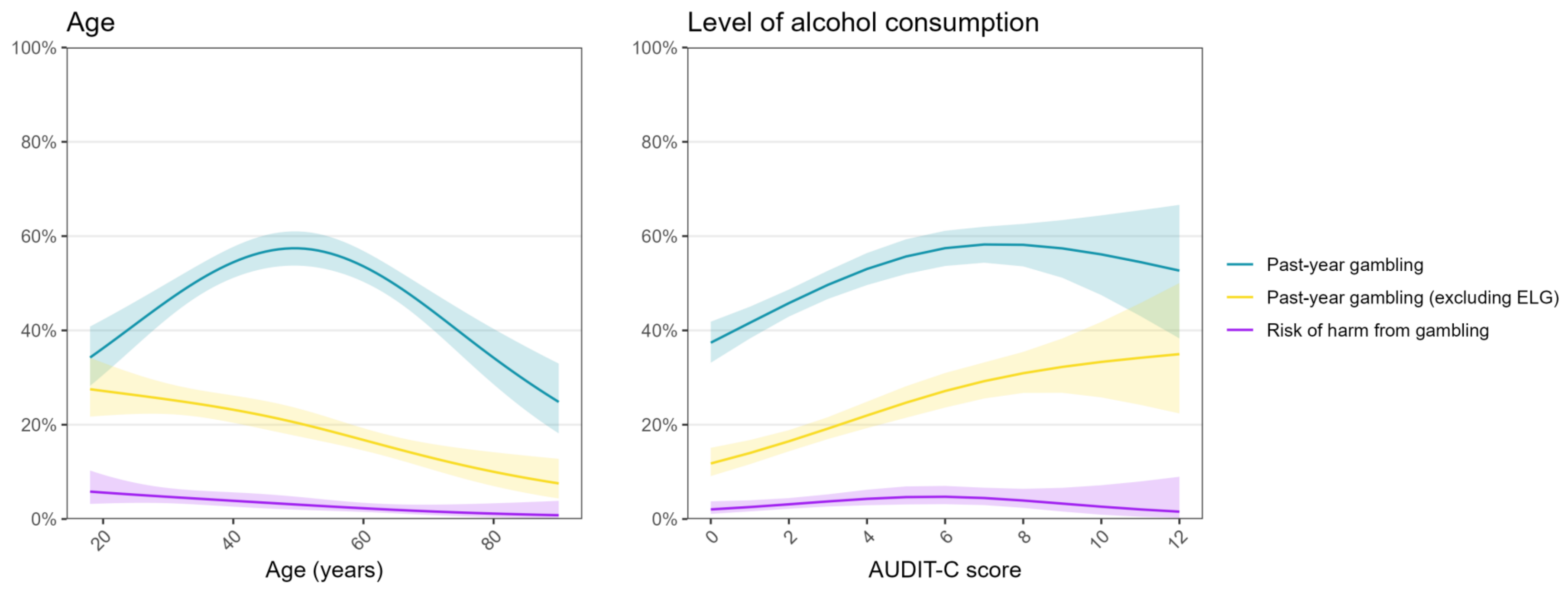

Figure S1. Prevalence of past-year gambling and risk of harm from gambling for all ages and AUDIT-C scores. Lines represent predicted estimates from logistic regression models with age/AUDIT-C modelled using restricted cubic splines. Shaded bands represent 95% confidence intervals. ELG, exclusive lottery gambling.

Table S3. Associations of risk of harm from gambling with past-month psychological distress – sensitivity analyses removing history of mental health conditions, smoking status, and level of alcohol consumption in turn from the fully adjusted model

	**Moderate/severe distress**		
	**Excluding mental health history, OR_{adj} [95%CI]**[1]	**Excluding smoking status, OR_{adj} [95%CI]**[2]	**Excluding alcohol consumption, OR_{adj} [95%CI]**[3]
**Risk of harm from gambling** (among adults)			
No	-	-	-
Yes	1.24 [0.60–2.55]	1.15 [0.58–2.30]	1.00 [0.50–1.99]
**Risk of harm from gambling** (among past-year gamblers, including ELG)			
No	-	-	-
Yes	1.40 [0.68–2.91]	1.33 [0.66–2.70]	1.30 [0.65–2.58]
**Risk of harm from gambling** (among past-year gamblers, excluding ELG)			
No	-	-	-
Yes	1.70 [0.72–4.03]	1.56 [0.63–3.83]	1.48 [0.63–3.45]

CI, confidence interval. ELG, exclusive lottery gambling. OR_{adj}, adjusted odds ratio.
[1] Estimates from logistic regression adjusted for age, gender, occupational social grade, children in the household, smoking status, and level of alcohol consumption.
[2] Estimates from logistic regression adjusted for age, gender, occupational social grade, children in the household, history of ≥1 mental health conditions, and level of alcohol consumption.
[3] Estimates from logistic regression adjusted for age, gender, occupational social grade, children in the household, history of ≥1 mental health conditions, and smoking status.
